# Interventions for Parental Anxiety in Preparation for Pediatric Surgery: A Narrative Review

**DOI:** 10.3390/children8111069

**Published:** 2021-11-20

**Authors:** Pooja Santapuram, Amanda L. Stone, Rachel Lane Walden, Louise Alexander

**Affiliations:** 1 School of Medicine, Vanderbilt University, Nashville, TN 37212, USA; 2 Department of Anesthesiology, Vanderbilt University Medical Center, Nashville, TN 37212, USA; amanda.l.stone@vumc.org; 3 Department of Pediatrics, Vanderbilt University Medical Center, Nashville, TN 37212, USA; 4 Annette and Irwin Eskind Family Biomedical Library, Vanderbilt University, Nashville, TN 37212, USA; rachel.l.walden@vanderbilt.edu

**Keywords:** preoperative anxiety, parent, children, anesthesia, perioperative

## Abstract

The preoperative experience can cause significant anxiety for both pediatric patients and their parents in the lead up to a surgical procedure. Pediatric anxiety in a preoperative setting has been shown to have significant negative downstream effects on the clinical outcomes of children and the healthcare system as a whole. Studies have found that preoperative parental anxiety has significant negative effects on children, regarding anxiety and emotional response. Therefore, interventions for parental preoperative anxiety are important to reduce the child’s anxiety. This review provides a brief overview of a broad range of strategies used to alleviate parental anxiety in a preoperative setting. Preoperative education, play-based interventions, music therapy, the presence of parents at induction of anesthesia, and integrative preoperative preparation programs have all demonstrated some evidence for reducing parental preoperative anxiety. The ultimate goal of using interventions for parental preoperative anxiety is to equip healthcare systems to better support families and optimize the perioperative outcomes of children.

## 1. Introduction

The preoperative surgical and anesthetic experience for many pediatric patients, as well as their parents, is stressful, and a common source of anxiety. Recent studies have indicated that up to 75% of children [1] and 74% of parents experience preoperative anxiety leading up to a pediatric surgical procedure [2]. Additionally, the presence of parental anxiety is known to affect the child’s anxiety [3,4].

Preoperative anxiety in pediatric populations, from clinical and healthcare system perspectives, has been linked to numerous downstream effects. Clinically, preoperative anxiety has been linked to increased postoperative delirium [5], increased postoperative pain [6], and a greater need for analgesia intraoperatively [6]. Further, preoperative anxiety has demonstrated associations with numerous maladaptive behaviors following discharge from the hospital, such as separation anxiety, sleep disruption, aggression, and bed wetting [5,7,8]. In terms of healthcare system consequences, preoperative anxiety has been associated with extended stays in recovery rooms, surgery delays, an increased need for postoperative care, and overall greater costs [5,9].

Although much of the literature on the impact of perioperative anxiety on clinical and healthcare systems focuses specifically on the child’s anxiety, preoperative parental anxiety appears to negatively impact a child’s emotional state and could be an important “target” for intervention. Specifically, a child’s anxiety level is shown to be highly associated with the parents’ anxiety levels during the preoperative period [3,4]. Furthermore, the anxiety parents experience prior to their child’s surgery has been identified as a major predictor of preoperative anxiety in children [7,10]. Studies have found significant associations between preoperative parental anxiety and a child’s anxiety, and behaviors in the postoperative period, even up to 6 months following surgery [11,12]. Preparing parents for surgery, i.e., by equipping them with the appropriate tools and knowledge to manage their own distress in front of their child, could reduce their anxiety levels [13].

A variety of factors could play a role in perioperative parental anxiety, including parental separation [14], lack of control over the environment, or the risk of complications or mortality [4]. Ayenew et al. [2] found that parental gender, fear of postoperative pain, the age of the child, and information about the anesthesia were predictive factors associated with preoperative parental anxiety. Additionally, there are multiple mechanisms potentially driving the interrelation between the parent and child’s perioperative anxiety. Shared genetic vulnerabilities, social learning theory (e.g., parental modeling and reinforcement), and shared environmental cues could all contribute to the relationship between the parent and child’s perioperative anxiety [15,16,17,18]. Thus, parental anxiety represents an important target for interventions aimed at alleviating a child’s anxiety. Unfortunately, a survey conducted in 2006 found that no treatment for preoperative anxiety has been consistently administered to the vast majority of families in the United States [19].

This narrative review examines the research on targeted interventions for preoperative parental anxiety, with a focus on strategies that demonstrate preliminary evidence for efficacy through positive outcomes. The literature search was performed in MEDLINE via the PubMed interface and Google Scholar for all papers published from inception to September 2021, using relevant keywords. Primary search terms, used in combination, included “parental anxiety” OR “parent preparation” OR “pediatric perioperative anxiety” OR “pediatric preoperative anxiety.” Papers were chosen to highlight themes and categories of interventions that clearly targeted parental anxiety with some evidence of anxiety reduction. Specifically, preoperative education, play-based interventions, music therapy, the presence of parents during induction of anesthesia (PPIA), and integrative preoperative preparation programs have all demonstrated some evidence at reducing parental preoperative anxiety, and will be discussed in the context of this review. Although a number of these interventions include the child (and additionally, measure the child’s anxiety), this review focuses on interventions that at least had a component that engaged parents. Table 1 summarizes the primary categories of interventions discussed and key findings.

## 2. Preoperative Anxiety Interventions

### 2.1. Preoperative Education

Preoperative education encompasses the use of formal or informal teaching or educational materials to prepare children and their families for an operation. Studies evaluating preoperative education, in a format that allows parents to express doubts and ask questions (e.g., educational counseling, informational preoperative visit), were found to reduce parental anxiety. Specifically, a randomized control trial comparing the efficacy of informational leaflets and counseling (i.e., clarification of doubts and reassurance of positive thoughts) to the standards of care for children undergoing surgery for congenital heart disease found a significant reduction in parental preoperative state anxiety, measured by the State Trait Anxiety Inventory (STAI), and a decrease in stress, measured by the Index of Clinical Stress (ICS) [20]. Similar findings were observed for parents of children undergoing ear, nose, and throat surgery, in which an informational visit during the preoperative period was shown to reduce parental anxiety by the Observational Scale of Behavioral Distress (OSBD-R) and STAI, as well as increased parental satisfaction of the treatment process through a postoperative treatment satisfaction questionnaire (PSQ-18). [21].

#### 2.1.1. Paper-Based

Interventions that provide paper-based educational materials alone without an interactive component have shown mixed efficacy. Studies have shown the effectiveness of educational booklets/leaflets objectively and subjectively, reducing maternal anxiety measured by STAI and in focus group discussions [13,22]. Conversely, other studies have not observed similar effects [23,24]. More studies on preoperative education, and the most acceptable and effective components of an educational intervention, are needed to better elucidate its role in reducing preoperative parental anxiety. Paper-based educational tools show promise, but the variation in the types of materials provided, delivery style, and surgical populations limit the ability to make conclusions regarding efficacy. Challenges with wide range distribution, as well as the inability to tailor the materials to specific parental and surgery needs, are also challenges faced with these types of interventions.

#### 2.1.2. Audiovisual

The use of audiovisual devices and tools has increased with advances in technology. A systematic review and meta-analysis performed by Chow and colleagues [25] assessed the effectiveness of audiovisual interventions aimed at reducing preoperative parental anxiety. These interventions included informational and psychoeducational videos, animated websites, and web-based apps. They found comprehensive informational videos with psychoeducational components that provided both education and teaching on coping skills or modeling behavior were most effective at reducing parental anxiety. Their findings also suggested that audiovisual interventions targeted to parents’ anxiety alone were more effective than those targeted to both parent and child [25]. This may have been due to the fact that parental programs specifically addressed parents’ questions and concerns to a greater depth than the videos aimed at parent and child anxiety in combination. It highlights the need for further research to be undertaken to discover what content would lead to the greatest impact from a clinical standpoint.

To date, research on audiovisual preoperative preparation has focused most on video-based tools. RCTs of at-home video preparation have shown reductions in caregiver anxiety associated with surgery [26,27]. Similarly, RCTs of video preparation at the hospital on the day of surgery have shown reductions in parental anxiety, shown through a statistically significant reduction in anxiety measured by the Amsterdam Preoperative Anxiety and Information Scale (APAIS) [28]. A recent systematic review concluded that video education appeared more effective than booklets, non-medical-related videos, or standard hospital procedures in reducing parental anxiety, even concluding that video preparation alone may provide sufficient information to manage preoperative anxiety in parents [29]. Overall, the current literature highlights preoperative informational and psychoeducational videos as an effective method of reducing parental anxiety. The most impactful delivery type, content, and timing of delivery has yet to be clearly defined, however. Delivery before the day of surgery has shown promise, consistently, when compared to the day of surgery.

Finally, the emergence of mobile applications and virtual reality has opened up new avenues for audiovisual education involving interactive elements. Ji et al. (2016) specifically studied the efficacy of the drawMD app, in which patients were shown a personalized drawing with text annotations and procedure-specific notes on an iPad to explain a procedure to the parents. They found that parental anxiety levels were significantly lower by APAIS, measured immediately after the education, as well as following their child’s operation [30].

Further, virtual reality technology has allowed for more immersive education. For example, one study assessing the effect of mirroring the display of a virtual reality hospital tour, in which parents could co-experience the virtual reality tour through a mirrored display of what the child experienced, found this mode of preparation was effective in reducing parental anxiety [31]. In contrast, a study that measured parental anxiety in response to a virtual reality tour targeted at the child alone did not see similar reductions [32]. Though there are limited data evaluating the use of app- or virtual reality-based education, preliminary evidence suggests these may reduce parental anxiety, especially when the parent is involved and incorporated into the experience.

### 2.2. Play

Both standard and therapeutic play during the preoperative period could also reduce parental perioperative anxiety. Therapeutic play refers to engaging children in activities that are appropriate for their age and simultaneously distract them from or help them prepare for their upcoming surgery or procedure (i.e., doll demonstration) [33,34]. Parents often engage in play-based interventions alongside their children, and studies have measured the effects of these interventions specifically on parental anxiety. A RCT conducted by He and colleagues [33] comparing therapeutic play to a standard pamphlet about the surgical procedure found both groups experienced decreases in parental anxiety, but differences between groups did not reach statistical significance. Qualitative interviews indicated that parents found the therapeutic play intervention helpful for reducing their anxiety and familiarizing their child with the equipment in the operating room. Another study assessed standard group play in which both the parent and child were involved in a game of Jenga, and saw significantly decreased parental anxiety in the group that participated in the activity compared to those that had routine preoperative preparation [35]. These studies suggest that allowing parents to partake in play-based activity alongside their child shows promise in easing their own level of anxiety prior to their child’s surgery. Further research should evaluate the effectiveness and cost of play-based interventions compared to or in addition to others (e.g., audiovisual education) for parental anxiety.

Clown therapy represents a specific form of play that is directed by a trained individual dressed up in colorful attire paired with humor, cheerfulness, and spontaneity to distract and reduce child and parent anxiety. Some studies have found reductions in parental anxiety and worries following the presence of clowns in the preoperative setting [36,37,38]. Importantly, a comprehensive systematic review and meta-analysis of randomized control trials by Sridharan and Sivaramakrishnan (2016) found that clown therapy significantly reduced preoperative STAI in parents [39]. The extent to which clowns engage parents in play could potentially account for differences between studies, but it has not been measured. As some parents may have an intense fear of clowns, the efficacy of this intervention likely depends on the acceptability of clowns.

In general, it appears that play-based interventions can play a significant role in reducing parental anxiety in the preoperative setting, with increased success observed in situations in which parents are involved in the activity. Successful reduction in parental anxiety was observed in several studies when parents were involved in standard play and clown therapy alongside their children.

### 2.3. Music

Studies have also assessed the use of music therapy in the preoperative setting. One study found that both passive (i.e., patient or family preferred music for relaxation) and active (i.e., patients given opportunity to select and play instruments) music therapy reduces preoperative caregiver anxiety as measured by short form STAI [40]. Similarly, a study using developmentally appropriate live music interventions (i.e., singing, songwriting, listening, instrument playing) preoperatively also reduced parental anxiety and improved subjective perception of the facility [41]. This study additionally demonstrated that music therapy played a role in improving parents’ perception of the facility and making parents feel that both they and their children benefitted from music therapy [41]. In both of these studies, the parents/caregivers participated alongside their children in the music therapy, again reinforcing the principle that active parent involvement in the intervention plays a role in decreasing their anxiety.

### 2.4. Parental Presence

Having parents present during anesthetic induction (i.e., allowing children to remain with parent/caregivers until under general anesthesia) is a technique used at some institutions to try to reduce preoperative anxiety experienced by children and families. It is a common non-pharmacologic perioperative anxiety intervention utilized in pediatric anesthesia. A few studies have specifically assessed the effects of parental presence during anesthetic induction on preoperative parental anxiety. Studies assessing parental perspectives of parental presence during induction have been fairly positive, with one study finding that parents feel their presence reduces their own anxiety [42]. In a study where parents were permitted to use conventional methods to relax their children during the induction, parents experienced significant anxiety reductions postoperatively but not preoperatively [43]. In contrast, one study assessing this effect did not identify any significant effects on self-reported anxiety immediately after induction. Instead, they found that parents present during induction exhibited an increase in their heart rate and skin conductance level [44], which is consistent with increased anxiety. This suggests that parental presence during induction could potentially increase parental anxiety acutely following induction. In most cases, parental presence does not appear to make an objective difference in parental anxiety [45]. However, as Antunes and Diogo [46] emphasized, if parental presence is utilized it should be structured in a way that is both safe and effective to yield positive outcomes for both the parents and the child.

### 2.5. Integrative Preparation Programs

Integrative preoperative preparation programs encompass a large variety of programs in which patients and their families receive an intervention with multiple components designed to increase comfort and knowledge in preparation for the patient’s surgical procedure. Examples of preparation programs include the use of operating room or hospital tours, child-life, or behavioral/psychological preparation teaching families various strategies to minimize anxiety, such as distraction.

Preoperative preparation programs that combine multiple components, such as play and tailored education (e.g., paper-based, audiovisual, or in-person hospital tours), could significantly reduce parental anxiety [47,48,49,50]. Comprehensive preparation programs have also resulted in greater parental satisfaction compared to receiving no standard preparation [47].

Anxiety-reduction, Distraction, Video modeling and education, Adding parents, No excessive reassurance, Coaching, and Exposure/shaping (ADVANCE), implemented and evaluated by Kain and colleagues [51], represents an example of a behavioral and psychological preoperative preparation program targeting parents. In one of the most rigorously designed RCT studies in this area, they compared ADVANCE to three other groups (parental presence, oral midazolam, and control), and found that the ADVANCE intervention resulted in the greatest reduction of parental anxiety in the preoperative holding area as well as after induction of anesthesia. This program was additionally found to be successful compared to other groups in lowering the incidence of emergence delirium, decreasing the analgesics required, and resulting in faster discharge times from the recovery room, suggesting that parent involvement and reduced parent anxiety could play a role in better postoperative outcomes for children.

Multiple features of preparation programs (e.g., timing, location, and extent of interaction) could impact the efficacy of the interventions. It is challenging to differentiate if the success is due to the combination of features or more specifically due to one feature alone. Further research is needed to evaluate which features are most helpful in order to optimize effectiveness. Additionally, programs incorporating the entire family demonstrate strong outcomes particularly from a cost–benefit perspective. Kain and colleagues found that the ADVANCE program could substantially save healthcare costs and time [51]. Though some may argue that such preparation programs have high costs at implementation, the potential impacts they could have at reducing hospital costs in the long-term, compared to the standard approach, are significant.

## 3. Discussion

It is widely recognized that pediatric surgery is a stressful and anxiety-provoking experience for parents in addition to children. Parents are intricately involved in the entire preoperative course for their children, and there is a strong concordance between parental and child anxiety preoperatively. High levels of preoperative anxiety in parent–child dyads could lead to a number of adverse clinical outcomes for the child (e.g., emergence delirium, higher postoperative pain) and impact healthcare systems through increasing the amount of time and personnel needed for care on the day of surgery. Proactively intervening on preoperative parental anxiety could improve outcomes for pediatric patients as well as improve functioning of the healthcare system. Due to the lack of consistent measurements and methodologies on this topic, this narrative review of the literature was undertaken to provide an overview of the current types on interventions implemented in this area. It is not intended to provide an evidence base for specific implementation of interventions.

Single interventions, including education, play, music, and parental presence for induction have all shown promise, but each has limitations and areas for further evaluation. Programs that integrate several different interventions across multiple timepoints in the perioperative period (e.g., week before surgery and day of surgery) may show greater efficacy. It is important, however, to understand which elements lead to the greatest improvements in parent and child outcomes to optimize their content and cost-effectiveness. As parents bring their own experiences, fears, and reactions to the pediatric surgical experience, interventions need to tailor content to specifically engage parents and not just the child. Consistent screening and risk stratification of preoperative anxiety in parents before the day of surgery could help guide personalization and implementation of interventions consistent with a precision medicine approach. Brief screening and risk stratification strategies have been developed for pediatric chronic pain populations [52] to better tailor intervention delivery and improve assessment of intervention effects. Developing a similar approach for preoperative anxiety could help identify families with the greatest intervention needs and allocate resources in the most cost-effective manner.

In summary, it is critical to the health of the child, parent, and healthcare system as a whole to implement interventions aimed at reducing parental anxiety about their child’s surgery. Parents are crucial partners for healthcare personnel throughout the perioperative period, and are often present with the child in the waiting area, preoperative holding room, and post-anesthesia care unit. Empowering and equipping parents to most effectively manage their own (and their child’s) anxiety could greatly improve care satisfaction for the family, child surgical outcomes, and the efficiency of care on the day of surgery.

**Table 1:** Summary of findings on interventions aimed at reducing preoperative parental anxiety.

Intervention	Characterized by	Findings
Education	Booklet/leaflet [13,20,22,23,24] Informational tour [21] Tablet [25,30] Videos (at-home vs. in preoperative setting) [25,26,27,28,29] Virtual reality [31,32]	Use of these educational materials showed some success in reducing parental anxiety. Educational videos showed efficacy when used prior to (and on the day of) surgery, and may even be more effective than other forms of educational interventions.
Play	Therapeutic play [33,34] Group play [35] Clown therapy [36,37,38,39]	These play-based interventions showed a significant reduction in parental anxiety, particularly when parents were also involved in the activity.
Music	Active music therapy [40,41] Passive music therapy [40]	Both active and passive music therapy interventions with parental involvement showed reductions in parental anxiety.
Parental Presence	Presence of parents during induction [42,43,44,45,46]	Findings were mixed. Parents reported subjective reductions in anxiety, but this might result in acute increases in anxiety immediately following induction.
Integrative Preparation Program	ADVANCE [51] Education + play [47,48,49,50]	Preparation programs simultaneously utilizing multiple interventions showed promise at reducing parental anxiety. More research is needed to determine the most effective components.
